# The simple production of nonsymmetric quaterpyridines through Kröhnke pyridine synthesis

**DOI:** 10.3762/bjoc.11.193

**Published:** 2015-09-30

**Authors:** Isabelle Sasaki, Jean-Claude Daran, Gérard Commenges

**Affiliations:** 1 CNRS, LCC (Laboratoire de Chimie de Coordination), 205 route de Narbonne, F-31077 Toulouse, France; 2 Université de Toulouse, UPS, INP, LCC, F-31077 Toulouse, France

**Keywords:** heterocycles, Kröhnke pyridine synthesis, Mannich bases, platinum, X-ray diffraction

## Abstract

Quaterpyridines have been demonstrated to be useful building blocks in metallo-supramolecular chemistry; however, their synthesis requires the preparation of sensitive building blocks. We present here three examples of nonsymmetric quaterpyridines that were easily obtained in yields of 70–85% by condensation of commercially available enones with 6-acetyl-2,2’:6’,2’’-terpyridine through a Kröhnke pyridine synthesis. Easy access to 6-acetyl-2,2’:6’,2’’-terpyridine starting from 2,6-diacetylpyridine and 2-acetylpyridine is described. The X-ray analysis of a chiral quaterpyridine and its Pt(II) complex is presented.

## Findings

Polypyridines have been demonstrated to be useful building blocks in metallo-supramolecular chemistry [1–3]. In particular, polypyridines joined at the 2,6-position have the ability to accommodate different coordination numbers preferred by a particular metal. These systems are of particular interest since they offer the possibility to control the assembly of helical systems to grid-like superstructures [1,4–8]. Depending on the metal ions, complexes with 2,2’:6’,2”:6”,2’’’-quaterpyridines as ligands can either adopt square planar, octahedral [3] or tetrahedral dinuclear double-helical geometries [9–10].

Different synthetic methods have been proposed for the preparation of these ligands but the first symmetric quaterpyridines were obtained by Kröhnke [11]. Constable et al. modified this methodology to prepare quaterpyridines bearing phenyl rings on the central pyridine units [7,12–13], then this method was further used by Grätzel et al. [14]. Potts et al. reported a high yield synthesis [15] that requires the cleavage of alkylthio-substituents before the final ligands can be obtained and precludes the preparation of asymmetrical analogues. Various coupling methodologies have also been developed: (i) the Ullmann reaction with copper powder at high temperature [9]; (ii) coupling of 6-halo-2,2’-bipyridines in the presence of nickel reagents [16–17], some of which are chiral [18–19]; and (iii) a Stille-based synthetic pathway [17,20], or a Suzuki–Miyaura cross-coupling reaction [21]. The use of triazine derivatives (which under particular conditions undergo an inverse Diels–Alder reaction) can also produce oligopyridines [22]. On the other hand, there are a limited number of reports dealing with the preparation of nonsymmetric quaterpyridines; Constable et al. proposed a multistep synthesis of 4’-(alkylthio)quaterpyridines [23] to avoid the Stille palladium-catalyzed coupling, whereas Fallahpour obtained the 4’-nitroquaterpyridine by employing the Stille coupling method [24]. Sauer et al. extended the use of triazine derivatives to the synthesis of 4-bromooligopyridines [25].

Constable et al. investigated the coordination behaviour of chiral 2,2’:6’,2”:6”,2”’-quaterpyridines bearing fused chiral groups in the 5,6- and 5”,6”-positions [26]. Recently, a synthesis strategy of asymmetrically substituted 2,2’:6’,2”:6”,2”’-quaterpyridines was described that required a multistep synthesis producing elaborate intermediates [24,27].

This short survey of the different ways to produce quaterpyridines (and in particular, nonsymmetric quaterpyridines) shows that a new simple synthesis can be quite relevant.

**Scheme 1:**
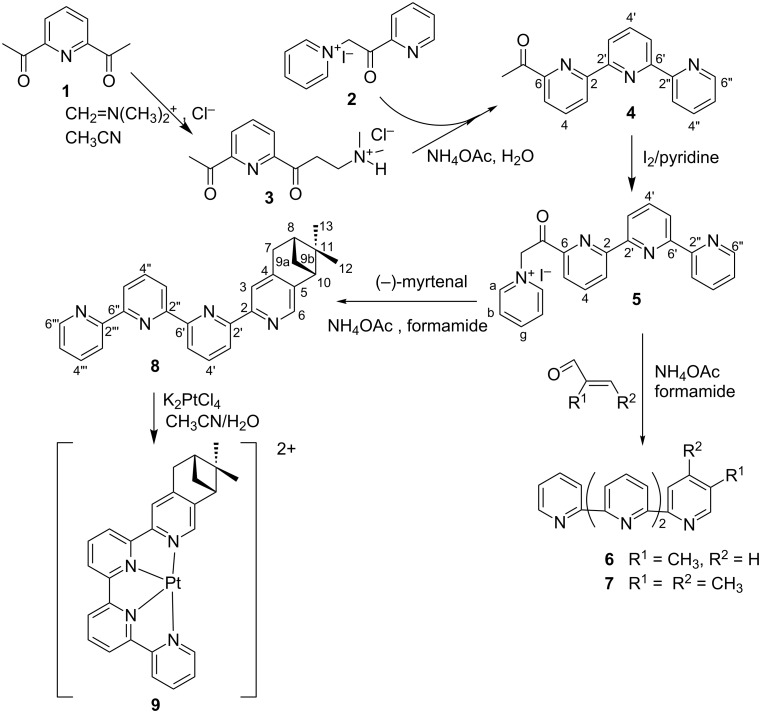
Synthesis of the quaterpyridines **6–8** and of the platinum complex **9**.

The synthetic approach adopted is based on the Kröhnke pyridine method [11]. The asymmetrical quaterpyridines were obtained in a four-step synthesis (Scheme 1).

Starting from 2,6-diacetylpyridine (**1**), it was possible to carry out the reaction with Eschenmoser’s salt on only one acetyl group leading to the nonsymmetric Mannich salt **3** as the masked enone. Pure compound **3** was only obtained in the presence of an excess of Eschenmoser’s salt, by addition of 2 equiv of (*N*,*N*-dimethyl)methyliminium chloride to **1**, in acetonitrile at room temperature. Then, compound **3** was condensed with *N*-[2-oxo-2-(2-pyridinyl)ethyl]pyridinium iodide (**2**) in the presence of ammonium acetate and gave 6-acetyl-2,2’:6’,2’’-terpyridine (**4**). This intermediate was cited by Potts [28] but to our knowledge, was not described. During the preparation of nonsymmetric quaterpyridines, 6-acetyl-2,2’:6’,2’’-terpyridine (**4**) is the key intermediate.

Recently Potvin et al. proposed a simple synthesis of 6,6”-diacetyl-4’-aryl-2,2’:6’,2”- terpyridines [29]. The 6,6”-diacetyl-4’-aryl-2,2’:6’,2”-terpyridine was obtained in a one-step synthesis from commercially available reactants in a 70% yield. Later, Solan et al. proposed a general strategy to prepare oligopyridylimine ligands using Stille-type coupling methodologies. They described different formyl or acetyl-oligopyridyl derivatives obtained by a Stille-type cross-coupling route requiring the preparation of stannylated derivatives [30], and namely, they described compound **4**.

After a further Kröhnke synthesis step leading to the corresponding acylpyridinium iodide **5**, the asymmetric ligands **6**–**8** were obtained by condensation with various α,β-unsaturated enones with good to excellent yield (see Supporting Information File 1 for the full experimental data). Von Zelewsky et al. showed that pinene-based chirality is easily introduced on the 2,2’-bipyridine or 2,2’:6’,2”:6”-terpyridine moieties [31–32]. In fact, the use of a chiral enone (i.e., (−)-myrtenal) led to the chiral 5,6-substituted quaterpyridine **8**. The corresponding platinum complex **9** was obtained by a classical synthesis, which entails the reaction of the platinum salt in the presence of the ligand in a mixture of acetonitrile and water under reflux for some hours (see Supporting Information File 1).

The quaterpyridines and the platinum complex **9** were fully characterized by NMR, EIMS, and elemental analysis. In particular, for **5**, **8** and **9**, the chemical shifts of all the signals were identified from two dimensional NMR COSY, HMBC and HSQC spectra. Whereas all the chemical shifts of the protons in the free ligand **8** were different, in the corresponding complex **9**, the chemical shifts of some protons and of some carbons were not discernible, and in particular, some in the aromatic region. The ^1^H spectrum of the complex showed a general deshielding varying from 0.8 to 0.2 ppm, where the most important values were observed on the aromatic protons (H5’’’ and H6) and the much lower values on the aliphatic protons. We observed a deshielding of the aromatic carbons from 2 to 29 ppm, whereas only weak shielding was observed on the aliphatic carbons. We noticed that the most affected carbons are those on the meta- or para-positions to the nitrogen atoms that are coordinated to the Pt atom. The most important deshielding instances are observed on C5 and C4, belonging to the aromatic ring substituted by the pinene moieties. These may impose some strain in the Pt complex, resulting in a slightly different electronic environment as compared to the other nonsubstituted aromatic rings.

**Figure 1:**
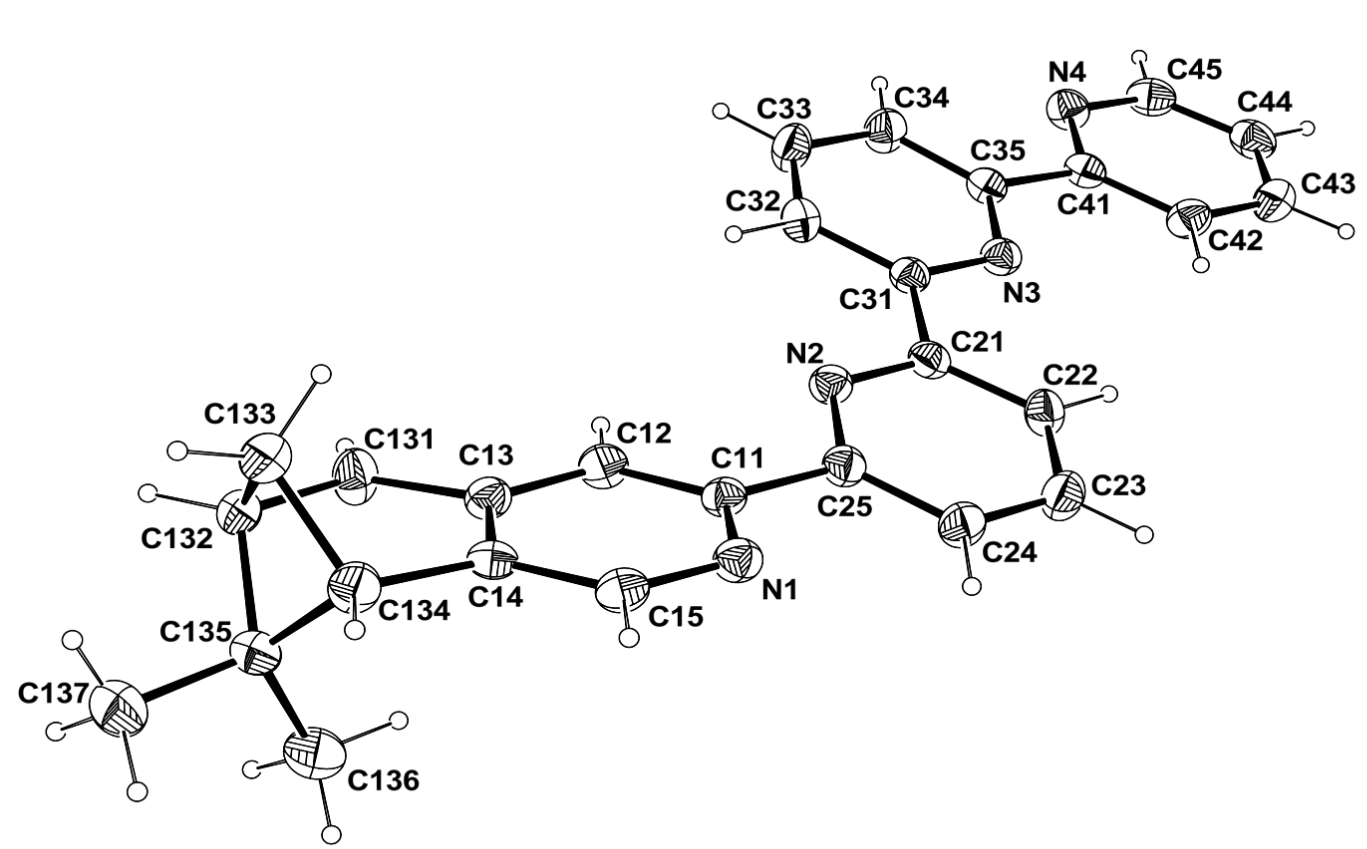
Molecular view of compound **8** with the atom labelling scheme. Ellipsoids are drawn at the 50% probability level. H atoms are represented as small circles of arbitrary radii.

**Figure 2:**
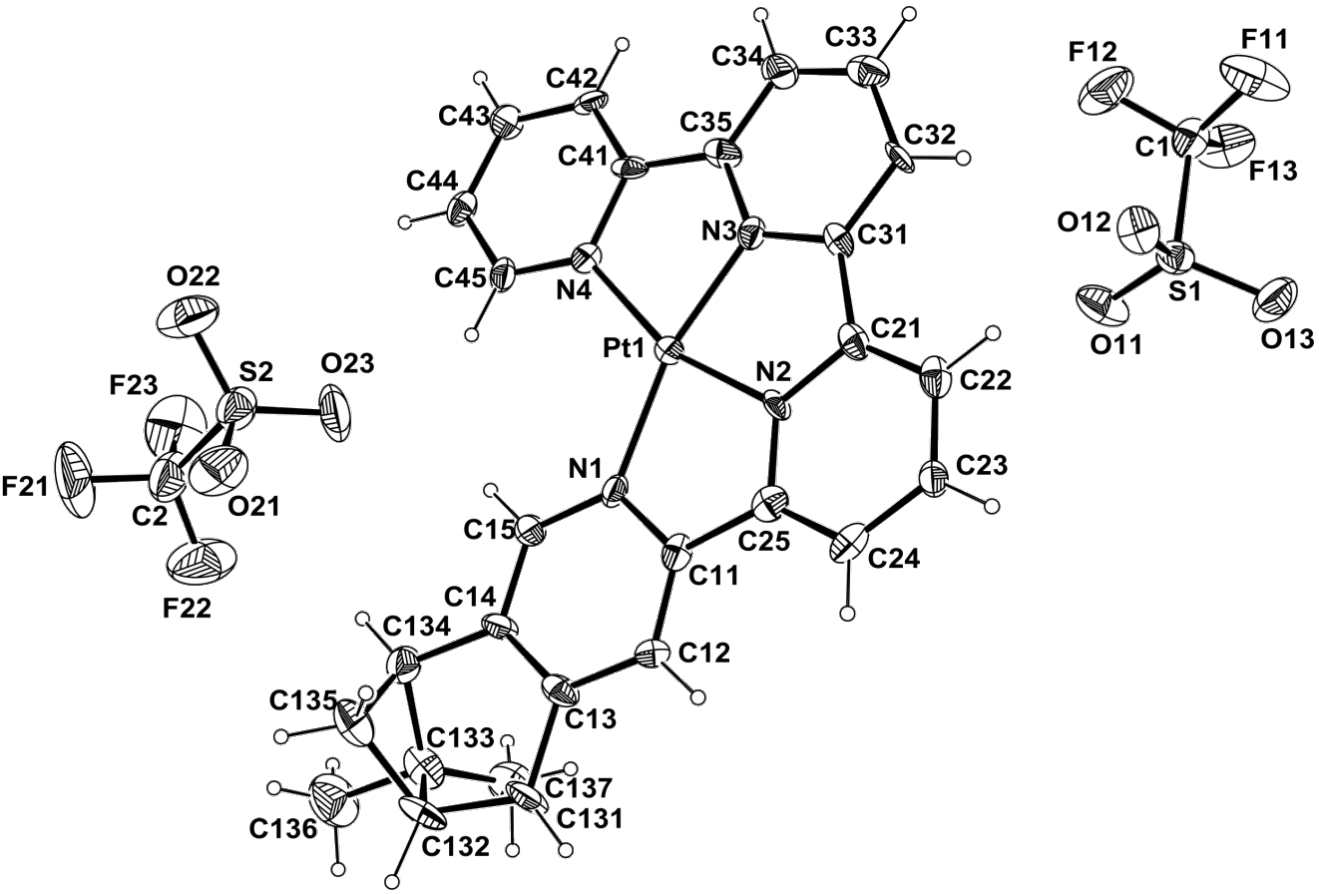
Molecular view of compound **9** using the atom labelling scheme. Selected values of bonds (Å) and angles (°) are given: Pt(1)-N(1): 2.046(6); Pt(1)-N(2): 1.936(7); Pt(1)-N(3): 1.934(6); Pt(1)-N(4):2.052(6); N(3)-Pt(1)-N(2): 82.2(3); N(3)-Pt(1)-N(4):80.3(3); N(3)-Pt(1)-N(1): 162.9(3); N(2)-Pt(1)-N(4):162.4(3); N(2)-Pt(1)-N(1): 80.8(3); N(1)-Pt(1)-N(4): 116.8(3). Ellipsoids are drawn at the 50% probability level. H atoms are represented as small circle of arbitrary radii.

Crystals of compounds **5**, **8** and **9** were obtained and their X-ray structures are described (for compound **5**, see Supporting Information File 1 for a description of the structure). The X-ray structures for the chiral quaterpyridine **8** and its Pt complex **9** are presented in Figure 1 and Figure 2 (for details, see Supporting Information File 1).

Compound **8** is comprised of 3 pyridine rings and a fused pyridine pinene fragment (Figure 1). The first 3 pyridine rings are roughly planar with the largest deviation from the mean plane being −0.052(2) at C33. The fourth pyridine ring and the first five atoms of the pinene part are also roughly planar with the largest deviation being −0.052(2) at C134. These two planes make a dihedral angle of 20.48(5)°. The carbon atoms C133 and C135 of the pinene fragment are roughly symmetrically distributed above and below the N1-C11-C12-C13-C14-C15-C131-C132-C134 planes by −1.118(3) and 1.008(3). Thus, both the C6 rings adopt a half-chair conformation. The packing is stabilised by van der Waals contacts and weak π–π interactions between symmetry related N2-C21-C22-C23-C24-C25 and N4-C41-C42-C43-C44-C45 pyridine rings (1+x, y, z) with a centroid-to-centroid distance of 3.761(2) Å, and an average interatomic distance between planes of 3.425(1) Å, resulting in a slippage of 1.54 Å. The absolute configuration is established by the occurrence of the known chirality of the starting material as (−)-(1*R*,5*S*)-myrtenal was used.

Crystals of complex **9** were obtained by slow evaporation of a solution of the compound with trifluoromethanesulfonate as the counter ion. The complex **9** results from the coordination of the chiral quaterpyridine on the Pt atom (Figure 2). The asymmetric unit contains two triflate anions to equilibrate the charges. The four nitrogen atoms of the quaterpyridine are coordinated to the platinum resulting in a distorted, square planar environment. The PtN_4_ framework is nearly planar with the largest deviations being −0.008(4) Å at the Pt atom. However, to accommodate the tetracoordination, the N-Pt-N angles range from 80.3(3)° to 116.8(3)°. Because of the presence of the Pt atom, the absolute configuration can be reliably determined by X-ray analysis [33], confirming the (*R*,*S*)-configuration on the pinene part.

## Conclusion

The primary result of this work was the development of a novel, simple, synthetic route for nonsymmetric quaterpyridines and circumventing the production of complex intermediates. This was accomplished through the simple synthesis of 6-acetyl-2,2’:6’,2”-terpyridine. After the preparation of the corresponding pyridinium salt, the subsequent condensation with enones led to the desired quaterpyridines. The methyl groups on the pyridine ring are potential grafting points, which can be used without modifying the chelating properties of the polydentate ligands.

## Supporting Information

File 1 Experimental procedures, characterisation data for all new compounds and X-ray analysis of compound **5**.
Crystallographic data (excluding structure factors) have been deposited with the Cambridge Crystallographic Data Centre as supplementary publication no. CCDC 1401819-1401821. Copies of the data can be obtained free of charge on application to the Director at CCDC, 12 Union Road, Cambridge CB2 1EZ, UK (FAX: (+44) 1223-336-033; email: deposit@ccdc.cam.ac.uk).
