# Supplementary material for: The simple production of nonsymmetric quaterpyridines through Kröhnke pyridine synthesis
Source: Beilstein J Org Chem. 2015 Sep 30;11:1781–5. doi: 10.3762/bjoc.11.193 (PMC4661014; doi:10.3762/bjoc.11.193)
Supplement: File 1 — Experimental procedures, characterisation data for all new compounds and X-ray analysis of compound 5. Crystallographic data (excluding structure factors) have been deposited with the Cambridge Crystallographic Data Centre as supplementary publication no. CCDC 1401819-1401821. Copies of the data can be obtained free of charge on application to the Director at CCDC, 12 Union Road, Cambridge CB2 1EZ, UK (FAX: (+44) 1223-336-033; email: deposit@ccdc.cam.ac.uk). [file Beilstein_J_Org_Chem-11-1781-s001.pdf]

Supporting Information

for

A crafty way to nonsymmetric quaterpyridines through Kröhnke pyridine syntheses

Isabelle Sasaki*[1,2], Jean-Claude Daran[1,2] and Gérard Commenges[1,2]

Address: [1]CNRS; LCC (Laboratoire de Chimie de Coordination) , 205 route de Narbonne, F-31077 Toulouse, France and [2]Université de Toulouse, UPS, INP, LCC, F-31077 Toulouse, France

Email: Isabelle Sasaki - isabelle.sasaki@lcc-toulouse.fr

* Corresponding author

Experimental procedures, characterisation data for all new compounds and X-ray views of compound 5

Synthesis

General: Solvents and reagents were used as obtained from commercial sources. Compound **2**, *N*-[2-oxo-2-(2-pyridyl)ethyl]pyridinium iodide, was prepared as previously described [1]. NMR spectra were recorded on a Bruker DPX 300 at 300 or 75 MHz for ^{1}H or ^{13}C, respectively. The spectra were referred to residual solvent peaks. FAB mass spectra were recorded with a quadripolar Nermag R 10-10 instrument using NBA as matrix. Elemental analyses were performed at LCC on a Perkin-Elmer 2400 Serie II. Column chromatography was performed with neutral alumina (Brockmann I) deactivated with 8% water.

Syntheses:

2-Acetyl,6-[*N*,*N*-dimethylamino-1-oxopropyl]pyridine hydrochloride (3)

To a solution of 2,6-diacetylpyridine (4.9g, 29 mmol) in acetonitrile (60 mL) was added (*N*,*N*-dimethyl)methyliminium chloride (Eschenmoser's chloride salt, 4.5 g, 48 mmol). The reaction mixture was stirred at room temperature for 4 days. The white precipitate was filtered off, rinsed with acetonitrile and dried under vacuum. Yield: 5.52 g, 72%.

^{1}H NMR ($CDCl_3$, 25°C) δ = 8.24(dd, 1H, *J* = 8, 1Hz); 8.19(dd, 1H, *J* = 7.9, 1Hz); 8.01(dd, 1H, *J* =7.7, 7.8Hz); 4.04(t, 2H, *J* =7.1 Hz); 3.52(quad., 2H, *J* =7Hz); 2.88(d, 6H, *J* =5Hz); 2.80(s, 3H). Analysis for $C_{12}H_{17}N_2O_2Cl$ + $0.5H_2O$ calcd. C 54.24, H 6.83, N 10.54; found C 54.34, H 6.49, N 10.84.
DCI-MS *m/z*: 221$[M]^+$.

6-Acetyl-2,2':6',2''-terpyridine (4) [2]

Compound **3** (2.56 g, 10 mmol) and compound **2** (3.27 g, 10 mmol) were dissolved in 100 mL of distilled water. After addition of ammonium acetate (3 g, 39 mmol), the mixture was heated to reflux for one night. After cooling down, the precipitate was filtered off, rinsed with water and dried under vacuum (1.81 g). The pure white compound was isolated after chromatography on alumina with 20% AcOEt in pentane. Yield: 1.06g, 39%.

^{1}H NMR ($CDCl_3$, 25°C): δ = 8.85 (dd, 1H, *J* = 7.8, 1.2Hz, H(5)); 8.74 (ddd, 1H, *J* = 4.8, 2.0, 0.8Hz, H(6")); 8.65 (dd, 1H, *J* = 8, 1.2Hz, H(3")); 8.60(dd, 1H, *J* = 7.8, 1.0Hz, H(3')); 8.52(dd, 1H, *J* = 7.9, 1.1Hz, H(5')); 8.10(dd, 1H, *J* = 7.7 and 1.2 Hz, H(3)); 8.03(t, 1H, *J* = 7.9Hz, H(4')); 8.02(t, 3H, *J* = 7.7Hz, H(4)); 7.90(ddd, 1H, *J* = 7.9, 7.5, 1.8Hz, H(4")), 7.38(ddd, 1H, *J* = 7.5, 4.8, 1.2Hz, H(5")), 2.89(s, 3H, CH_3); ^{13}C{^{1}H }($CDCl_3$, 25°C): δ = 200.78(C=O); 156.45(C(2")); 155.90(C(6')); 155.80(C(2')); 154.97(C(2)); 153.34(C(6)); 149.62(C(6")); 138.40(C(4')); 138.15(C(4)); 137.32(C(4")); 124.75(C(5)); 124.30(C(5")); 121.90(C(3)); 121.82(C(3')); 121.56(C(3")); 121.44(C(5')); 26.21(CH_3).
Analysis for $C_{17}H_{13}N_3O$ calcd. C: 74.17; H: 4.76 ; N: 15.26; found C: 73.89; H: 4.56; N: 15.15. EI-MS *m/z*: 275 $[M]^+$.

***N*-[2-Oxo-2-(6-terpyridinyl)ethyl]pyridinium iodide (5)**

Acetyl terpyridine **4** (1.66 g, 6 mmol) and iodine (1.57 g, 6.2 mmol) were heated in 5 mL of dry pyridine at 110 °C for 4 hours. After cooling down, the beige precipitate was filtered off, rinsed with a few millilitres of cold pyridine and with ether. Yield: 2.23g, 77%.

^1H NMR (DMSO-d_6, 25°C): δ = 9.35(m, 2H, Hα), 9.11(dd, 1H, J = 7.9, 1.8Hz, H(5)), 8.96(mt, 1H, Hγ), 8.78(m, 1H, H(3')), 8.77(m, 1H, H(3")), 8.76(m, 1H, H(6")), 8.64(dd, 1H, J = 7.9, 0.9Hz, H(5')), 8.50(dd, 2H, J = 7.8and 6.8 Hz, Hβ), 8.36(t, 1H, J = 7.8Hz, H(4)), 8.21(dd, 1H, J = 7.6, 1Hz, H(3)), 8.19(t, 1H, J = 7.8Hz, H(4')), 8.04(m, 1H, H(4")), 7.52(ddd, 1H, J = 7.4, 4.7, 1.2Hz, H(5")), 7.11(s, 2H, CH_2); $^{13}C\{^1H\}$(DMSO-d_6, 25°C): δ = 205.75(C=0), 156.43 (C6'), 156.3(C2), 156.1(C2"), 154.2(C2'), 150.67 (C6), 149.83 (C6"), 147.22(Cγ), 147.08 (Cα), 139.64(C4), 138.81(C4'), 137.53(C4"), 128.66(Cβ), 126.31(C5), 124.77(C5"), 122.59(C3), 122.03 (C5'), 121.58(C3"), 121.19 (C3'), 67.67(CH_2) Analysis for $C_{22}H_{17}N_4OI$ calcd. C: 55.02 ; H: 3.57; N: 11.66 ; found C: 54.81; H: 3.02; N: 11.43. FAB-MS(mNBA) *m/z*: 353$[M-I]^+$

Quaterpyridines

Typical synthesis: Pyridinium salt **5** (240 mg, 0.5 mmol) and ammonium acetate (150 mg, 2 mmol) were suspended in formamide (2 mL). After the addition of the enone in excess (3 equiv) and heating at 80 °C, solubilisation was observed, then a precipitate appeared soon. Heating was maintained overnight. After cooling down, the precipitate was filtered off, rinsed with distilled water and dried under vacuum.

5-Methyl-2,2':6',2":6",2"'-quaterpyridine (6)

With metacroleine gives a beige powder. Yield: 138 mg, 85%; mp = 188-9°C

^1H NMR ($CDCl_3$, 25°C): δ = 8.62-8.72(m, 4H); 8.55(d, J = 5.5 Hz, 1H); 8.53 (s, 1H); 8.45(dt, J = 7.4 and 0.7Hz, 2H); 7.95-8.03(m, 2H) ; 7.88(dt, J = 7.8 and 1.8Hz, 1H); 7.68(dd, J = 7.7 and 1.5 Hz, 1H); 7.34(ddd, J = 7.4; 4.7 and 1 Hz, 1H); 2.42(s, 3H); $^{13}C\{^1H\}$($CDCl_3$, 25°C): δ = 156.24, 155.46, 155.36, 155.30, 153.67, 149.48, 149.08, 137.76, 137.48, 136.87, 123.75, 121.19, 121.06, 120.93, 120.72, 18.41.

Analysis for $C_{21}H_{16}N_40.3H_20$ calcd. C: 76.67; H: 5.09 ; N: 17.03; found C: 76.62; H: 4.92; N: 16.95. EI-MS *m/z*: 324$[M]^+$

4,5-Dimethyl-2,2':6',2":6",2"'-quaterpyridine (7)

With tiglic aldehyde gives a beige powder. Yield: 140 mg, 83%; mp = 176-7°C

^1H NMR ($CDCl_3$, 25°C) δ = 8.65-8.71(m, 3H); 8.63(dt, J = 5 and 0.8Hz, 1H); 8.39-8.48(m, 4H); 8.03(t, J = 8Hz, 2H); 7.85(dt, J = 7.8 and 1.8Hz, 1H) ; 7.32(ddd, J = 7.4; 4.7 and 1 Hz,

1H); 2.42(s, 3H); 2.32(s, 3H); $^{13}C\{^1H\}$ ($CDCl_3$, 25°C) δ = 156.22, 155.55, 155.47, 155.24, 153.96, 149.39, 146.53, 137.74, 137.65, 136.83, 132.61, 123.72, 121.90, 121.18, 121.06, 120.90, 120.78, 120.65, 19.51, 16.37.

Analysis for $C_{22}H_{18}N_4$ calcd. C: 78.08; H: 5.36; N: 16.56; found C: 77.56; H: 5.09; N: 16.41.

EI-MS *m/z*: 338$[M]^+$

4,5-[(1*R*,5*S*)-(Pineno)]-2,2':6',2":6",2"'-quaterpyridine (8)

With myrtenal gives a pale yellow powder. Yield: 142mg, 70%; mp = 163-4°C.

After cooling down, the brown paste is filtered off, rinsed with water and purified by chromatography with 10%AcOEt in pentane.

^1H NMR ($CDCl_3$, 25°C): δ = 8.72 (ddd, *J* = 4.8, 2, 1.2Hz, 1H, , H6"'), 8.70 (dd, *J* = 8, 1.2Hz, 1H, H3"), 8.67 (dt, *J* = 8, 1.2Hz, 1H, H3"'), 8.64 (dd, *J* = 8, 1.2Hz, 1H, H3'), 8.49 (dd, *J* =8, 1.2Hz, 1H, H5"), 8.46 (dd, *J* = 8, 1.2Hz, 1H, H5'), 8.45 (d, ^4J = 1Hz, 1H, H3), 8.26 (d, *J* = 0.5Hz,1H, H6), 8.08 (t, ^3J = 8Hz, 1H, H4"), 7.98 (t, ^3J = 8Hz,1H, H4'), 7.87(td, *J* = 7.6, 1.6Hz, 1H, H4"'), 7.34(ddd, *J* = 7.6, 4.8, 1.2Hz, 1H, H5"'), 3.14(d, *J* = 1.7Hz, 2H, H7), 2.91(t, ^3J = 5.6Hz,1H, H10), 2.74(dt, *J* = 9.6, 5.6Hz,1H, H9b), 2.36(msept, *J* = 2.8Hz, 1H, H8), 1.45(s, 3H, Me12), 1.28(d, *J* = 9.6Hz, 1H, H9a), 0.71(s, 3H, Me13); $^{13}C\{^1H\}$($CDCl_3$, 25°C): δ = 156.7(C2"'), 156.3(C2'), 156.0(C6'), 155.7(C6" or C2"), 155.6(C2" or C6"), 154.9(C2), 149.5(C6"'), 145.9(C6), 145.8(C4), 143.5(C5), 138.2(C4"), 138.1(C4'), 137.3(C4"'), 124.2(C5"'), 121.6(C3"'), 121.5(C3"), 121.3(C5"), 121.2(C3'), 121.0(C5'), 120.9(C3), 44.9(C10), 40.5(C8), 39.7(C11), 33.5(C7), 32.3(C9), 26.4(C12), 21.8(C13).

Analysis for $C_{27}H_{24}N_4$ calcd. C: 80.17; H: 5.98 ; N: 13.85 ; found C: 80.30; H: 6.12; N: 13.50. DCI-MS *m/z*: 405 $[MH]^+$. $[\alpha]_{546}$ = -90 (c = 1mg in 2mL $CHCl_3$).

Pt complex 9

Quaterpyridine **8** (53mg, 0.13 mmol) and K_2PtCl_4 (75 mg, 0.18 mmol) were refluxed in acetonitrile/H_2O 1:1 (20 mL) for 36 hours. After cooling down, the reaction mixture was filtered off and an excess of NH_4PF_6 (0.1 M) in water was added to the filtrate. The yellow-green precipitate was filtered off, rinsed with distilled H_2O then CH_2Cl_2 to obtain 100 mg (83%) of the product.

^1H NMR (DMSO-d_6, 25°C): δ = 9.24 (d, ^3J = 5.4Hz, 1H, H6"'), 8.75 (ABC syst., 1H, H3"'), 8.74 (s, 1H, H6), 8.70-8.54 (m, 6H, H5' ,4" ,3" , 5", 4', 3'), 8.63 (m, 1H, H3), 8.61(m, 1H, H4"'), 8.09(ABC syst., 1H, H5"'), 3.36(s, 2H, H7), 3.33(t, ^3J = 11.5Hz, 1H, H10), 2.86(ddd, *J* = 10.8, 5.9, 5.6Hz, 1H, , H9b), 2.42(m, 1H, H8), 1.49(s, 3H, Me12), 1.26(d, 1H, ^3J =10Hz,

H9a), 0.71(s, 3H, Me13) $^{13}C\{^1H\}$(DMSO-d_6, 25°C): δ = 159.31(C2'''), 157.32, 157.14, 156.96, 156.87, 156.80(C2', C6', C6'' , C2'' or C2), 155.02(C6'''),154.09(C4), 149.95(C5), 149.88 (C6), 145.15 and 145.12(C4 , C4'), 144.50(C4'''), 130.20(C5'''), 127.42(C3'''),126.92(C3), 126.70, 126.29, 125.98, 125.38(C3'' , C5'' , C3', C5'), 44.68 (C10), 39.78(C8), 39.23 (C11), 34.19(C7), 31.13 (C9), 26.23 (C12), 22.15(C13).

Analysis for $C_{27}H_{24}N_4Pt(PF_6)_2$. $2H_2O$ calc. C: 35.00; H: 3.13; N: 6.05; found C: 34.78; H: 2.77; N: 5.99. FAB-MS *m/z*: 744$[M-1PF_6]^+$ and 599$[M-2PF_6]^+$.

Triflate salt: Instead of NH_4PF_6, 90 mg (0.35 mmol) of $AgOSO_2CF_3$ was added and the reaction was heated to 70 °C for 30 min. After cooling down, the grey precipitate was filtered off. The next day the formed yellow precipitate was filtered of to yield 90 mg of the complex. Slow evaporation of the filtrate yielded crystals suitable for X-ray diffraction analysis.

Analysis for $C_{27}H_{24}N_4Pt(CF_3SO_3)_2.2H_2O$ calc. C: 37.26 ; H: 3.02 ; N: 5.99; found : C: 37.14; H: 2.32; N: 5.99.

Compound **4**

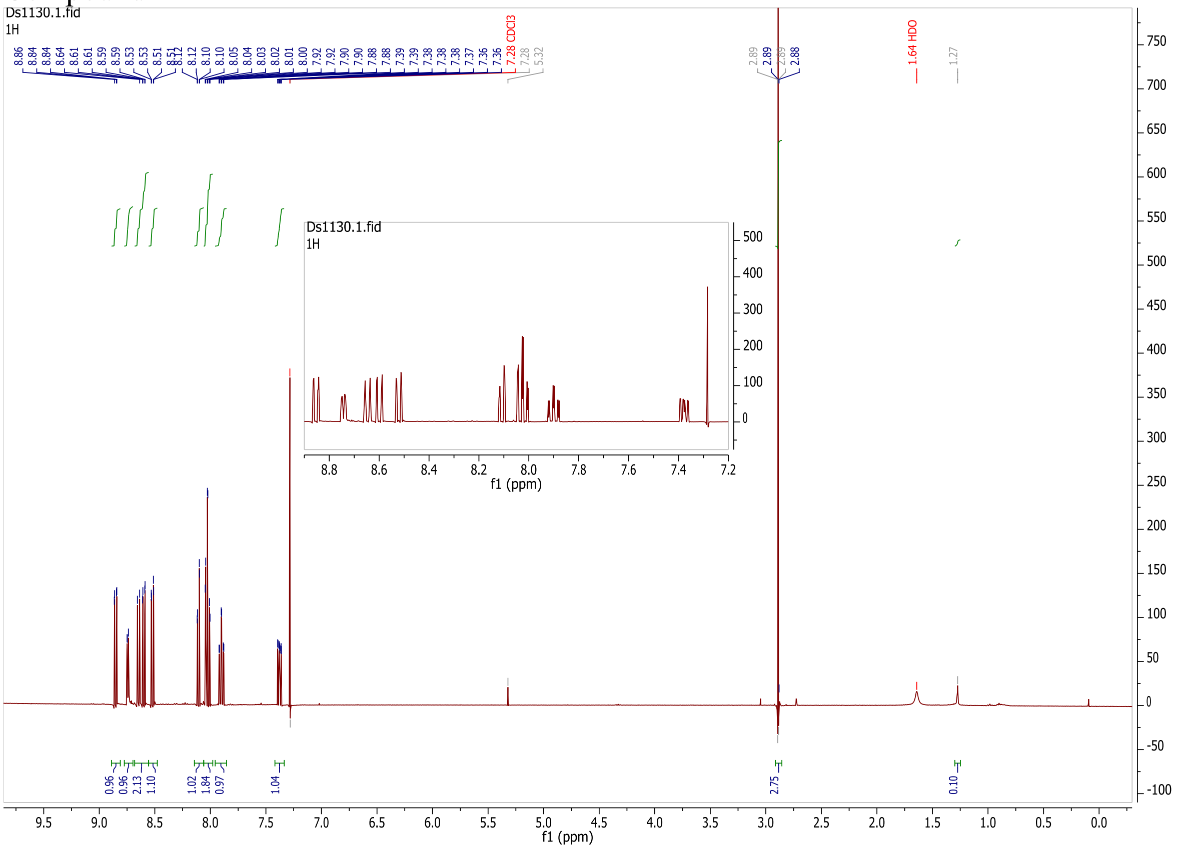

Ds1130.1.fid
1H
8.86
8.84
8.84
8.64
8.61
8.61
8.59
8.59
8.53
8.53
8.51
8.51
8.12
8.12
8.10
8.10
8.05
8.04
8.03
8.02
8.01
8.00
7.92
7.92
7.90
7.90
7.88
7.88
7.39
7.39
7.38
7.38
7.38
7.37
7.36
7.28 CDCl3
7.28
5.32
2.89
2.89
2.89
2.88
1.64 HDO
1.27
0.96
0.96
2.13
1.10
1.02
1.84
0.97
1.04
2.75
0.10
f1 (ppm)
Ds1130.1.fid
1H
f1 (ppm)

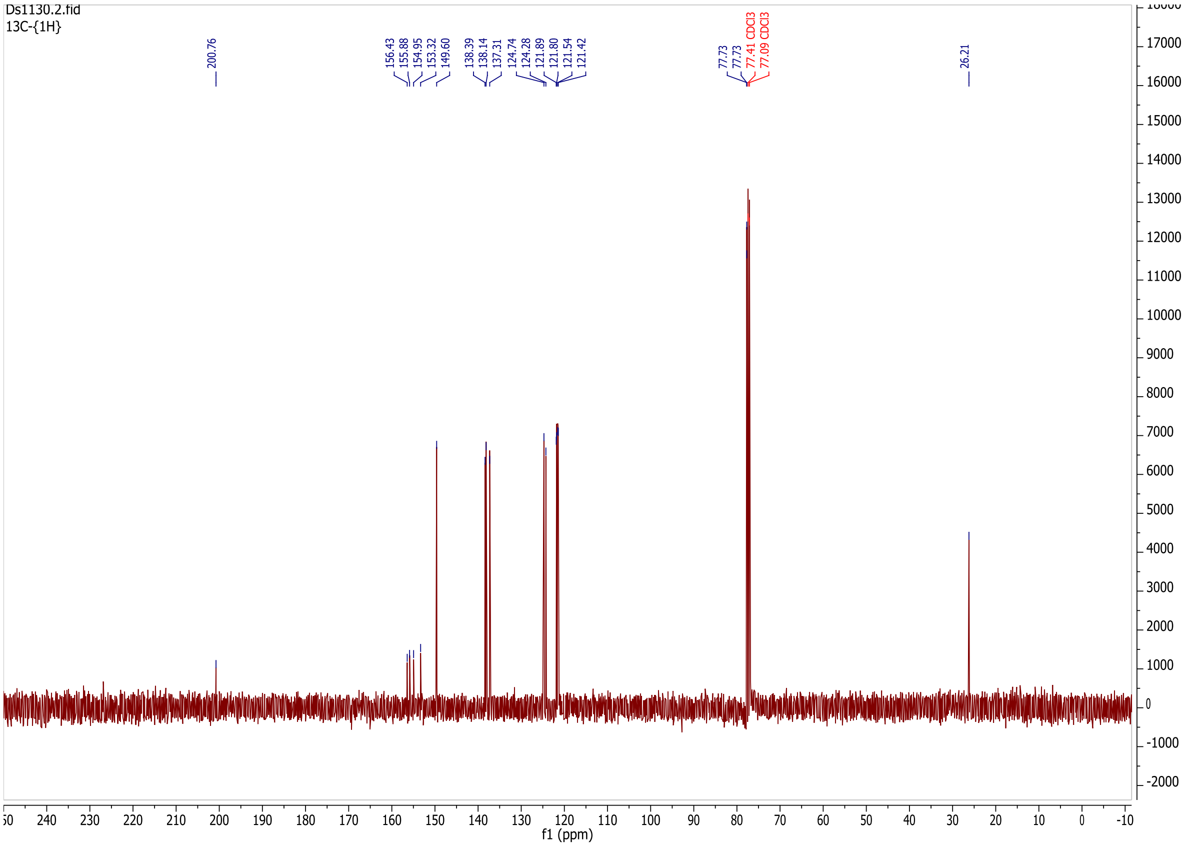

Ds1130.2.fid
13C-{1H}
200.76
156.43
155.88
154.95
153.32
149.60
138.39
138.14
137.31
124.74
124.28
121.89
121.80
121.54
121.42
77.73
77.73
77.41 CDCl3
77.09 CDCl3
26.21
f1 (ppm)

Compound **8**

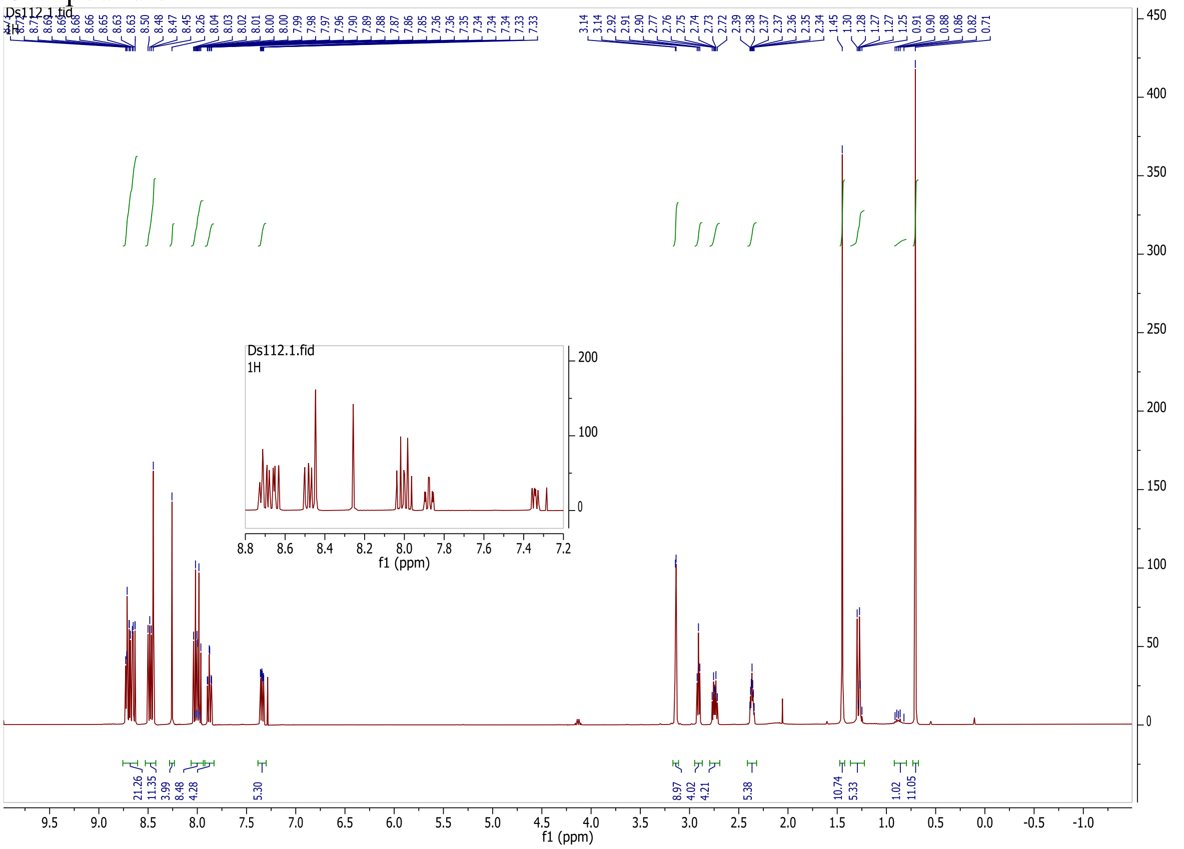

Ds112.1.fid
1H
f1 (ppm)

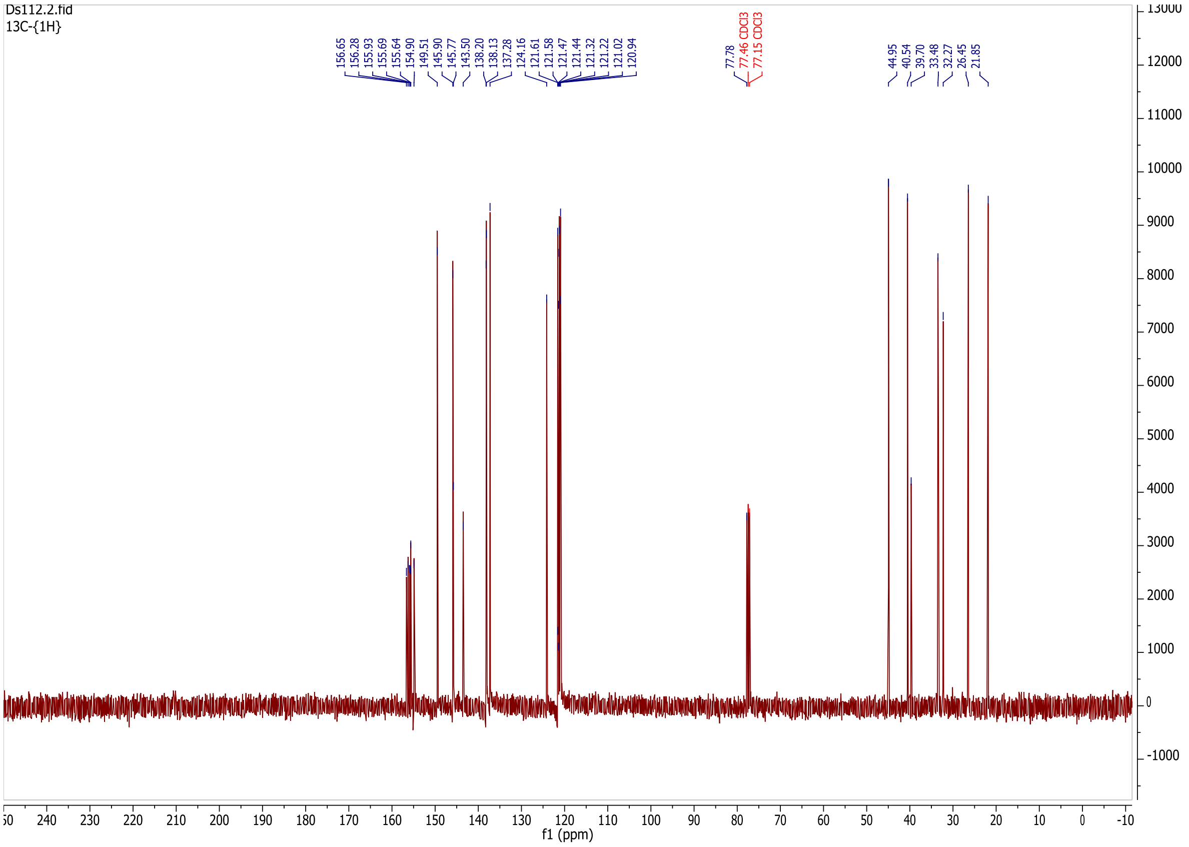

Ds112.2.fid
13C-{1H}
156.65
156.28
155.93
155.69
155.64
154.90
149.51
145.90
145.77
143.50
138.20
138.13
137.28
124.16
121.61
121.58
121.47
121.44
121.32
121.22
121.02
120.94
77.78
77.46 CDCl3
77.15 CDCl3
44.95
40.54
39.70
33.48
32.27
26.45
21.85
f1 (ppm)

Compound **9**

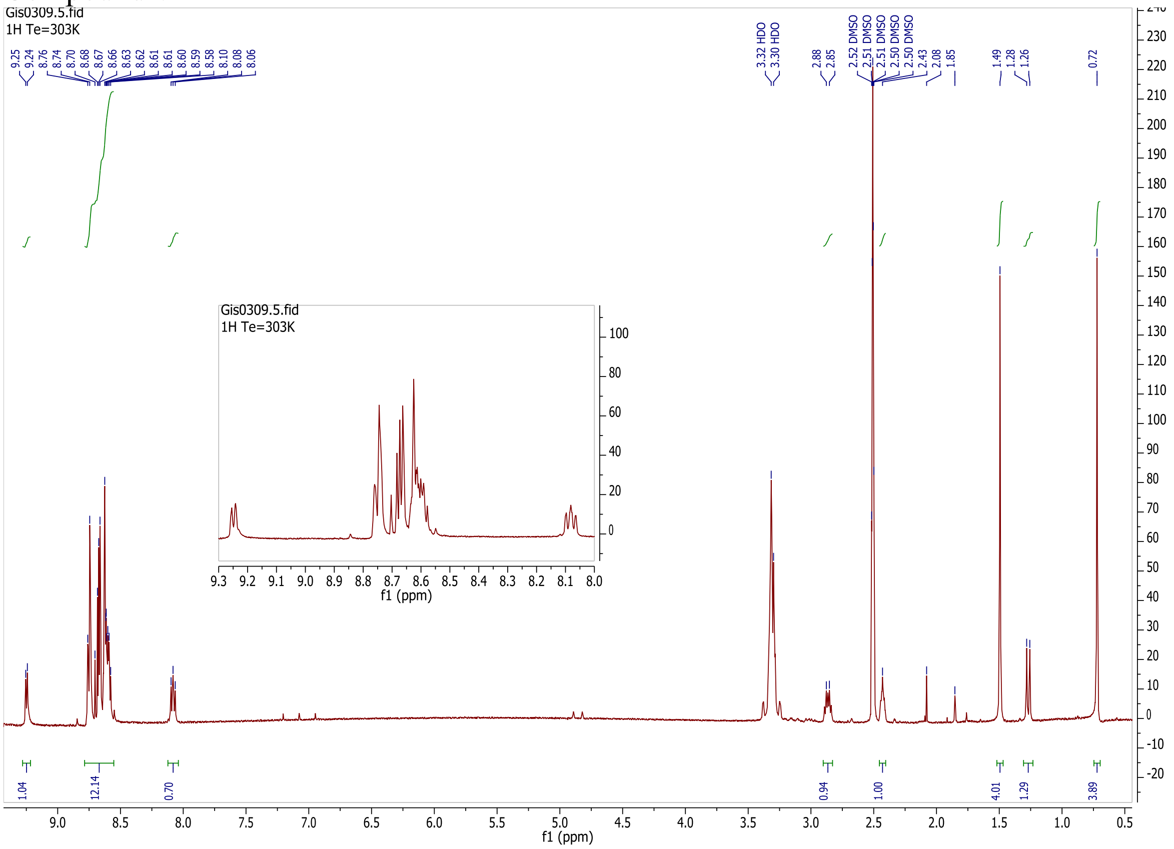

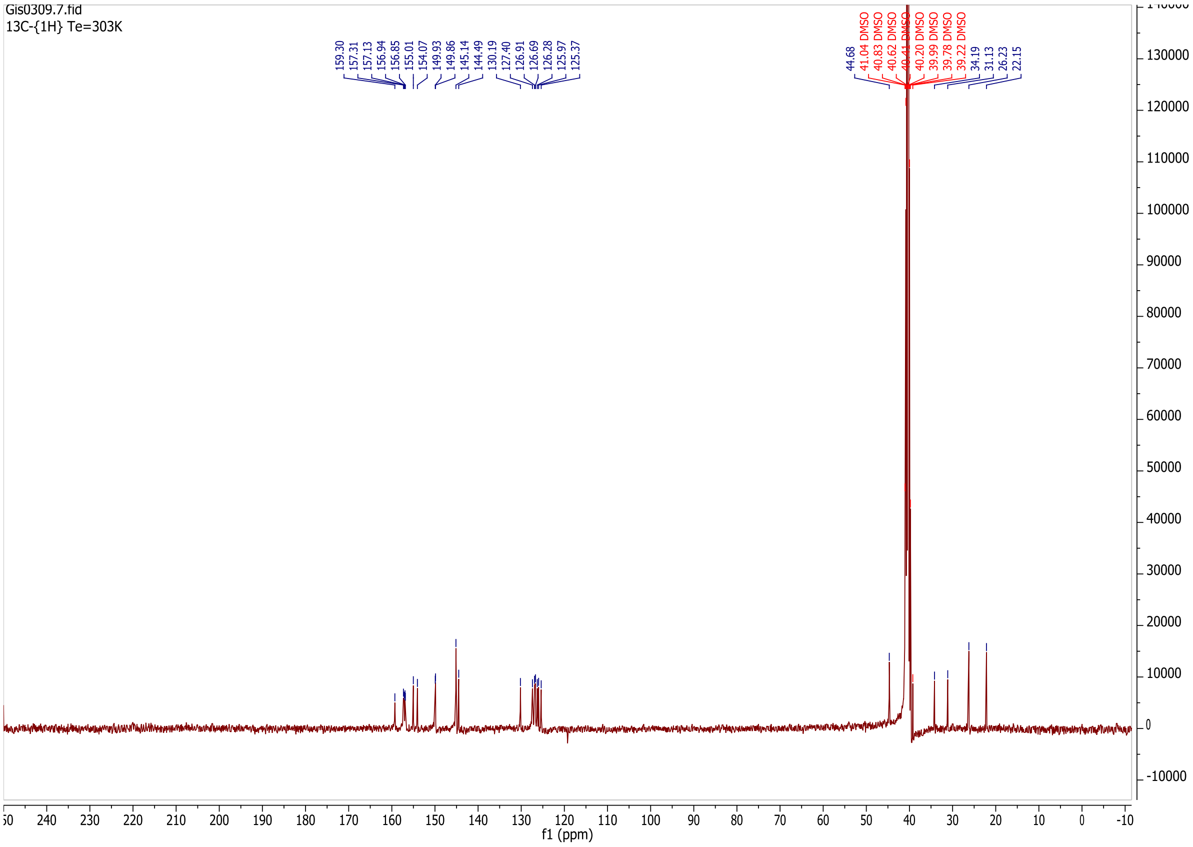

References for Synthesis

1. Kröhnke F., Gross F.K. *Chem. Ber.*, **1959**, *92*, 24-36.
2. Champouret Y. D.M., Chaggar R.K., Dadhiwala I., Fawcett J., Solan G. A., *Tetrahedron*, **2006**, *62*, 79-89.

X-ray analysis

The single crystal of each compound was mounted under inert perfluoropolyether at the tip of a glass fibre and cooled in the cryostream of an Oxford-Diffraction XCALIBUR CCD diffractometer.

The structures were solved by direct methods (SIR97 [1]) and refined by least-squares procedures on F^2 using SHELXL-97 [2]. All H atoms attached to carbon were introduced in calculation in idealised positions and treated as riding models. The drawing of the molecules was realised with the help of ORTEP32 [3,4]. Crystal data and refinement parameters are shown in Table S1.

Crystallographic data (excluding structure factors) have been deposited with the Cambridge Crystallographic Data Centre as supplementary publication no. CCDC 1401819-1401821. Copies of the data can be obtained free of charge on application to the Director, CCDC, 12 Union Road, Cambridge CB2 1EZ, UK (fax: (+44) 1223-336-033; e-mail: deposit@ccdc.cam.ac.uk).

Compound **5** is built up from 3 pyridine rings linked to a pyridinium through a CH_2CO fragment (Figure S1). The counter ion is iodine and the unit cell contains 4 I2 molecules. There are weak C-H···O hydrogen bond interactions linking two molecules through R22(16) graph set motif (Figure S2) [5,6]. There are also weak π–π interactions between symmetry related N4 C41 C42 C43 C44 C45 pyridine rings (1/2−x,1/2−y,−z) with a centroid to centroid distance of 3.619(2) Å, an interatomic distance between planes of 3.309(2) Å resulting in a slippage of 1.465 Å.

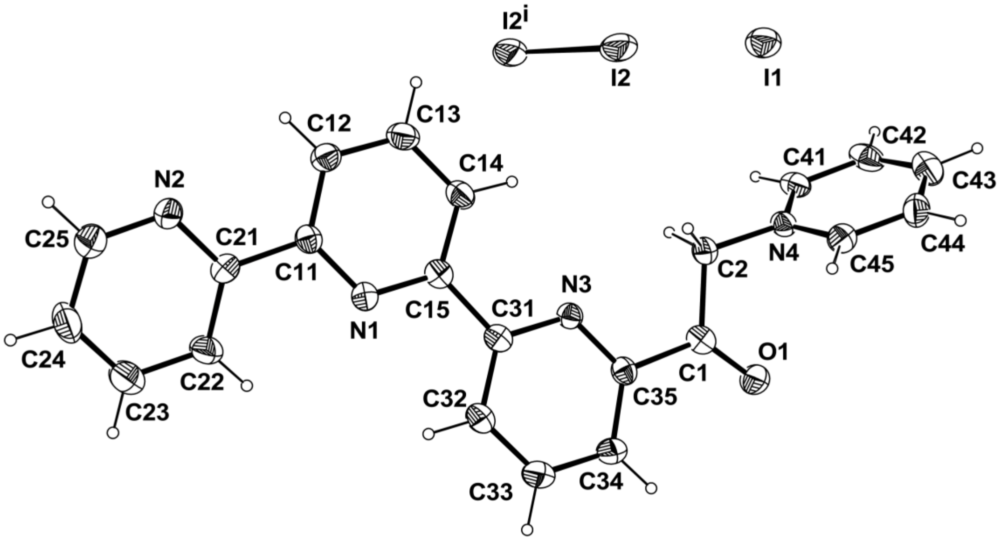

Figure S1: ORTEP view of compound **5** with the atom labelling scheme. Ellipsoids are drawn at the 50% probability level. H atoms are represented as small circle of arbitrary radii. [Symmetry code: (i) 1−x, y, 1/2−z].

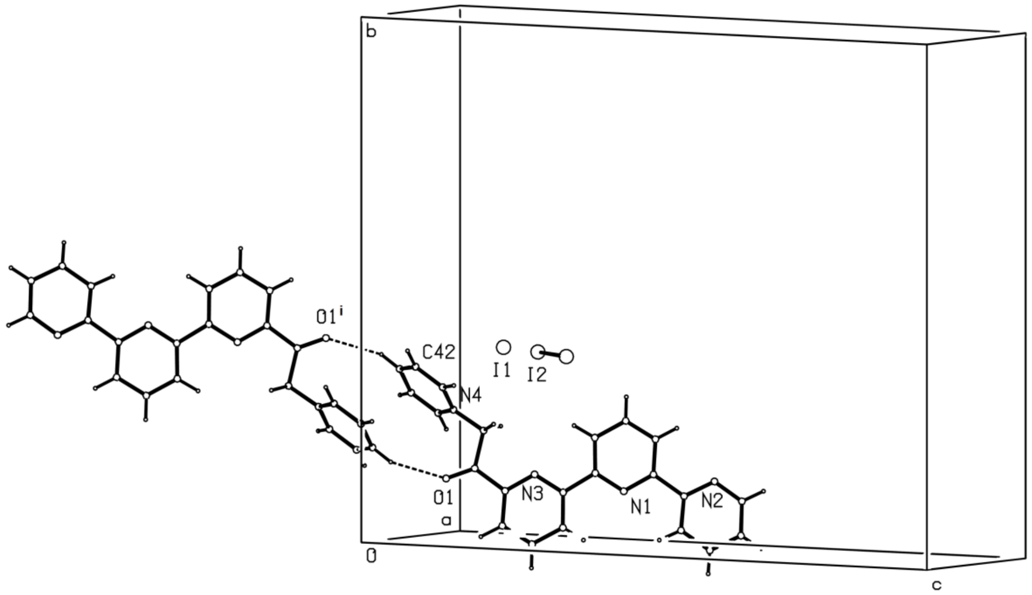

Figure S2: Partial packing view showing the formation of the $R^2_2(16)$ graph set motif. Hydrogen bonds are shown as dashed lines. [Symmetry code: (i) 1/2−x,1/2−y,−z].

Table S1: Crystal data for compounds **5**, **8** and **9**.

Identification code	**5**	**8**	**9**
Empirical formula	$C_{22}H_{17}N_4O, I_2$	$C_{27}H_{23}N_4$	$C_{27}H_{23}N_4Pt, (CF_3SO_3)_2$
Formula weight	607.20	403.49	896.72
Temperature, K	180(2)	180(2)	180(2)
Wavelength, Å	0.71073	0.71073	0.71073
Crystal system	Monoclinic	Orthorhombic	Orthorhombic
Space group	C2/c	$P2_12_12_1$	$P2_12_12_1$
a, Å	7.5692(6)	6.6689(3)	6.0628(2)
b, Å	20.8334(10)	14.8801(6)	9.2952(3)
c, Å	27.2051(13)	20.7775(9)	53.0543(17)
α, °	90.0	90	90.0
β, °	93.855(5)	90	90.0
γ, °	90.0	90	90.0
Volume, $Å^3$	4280.3(4)	2061.83(15)	2989.87(17)
Z	8	4	4
Density (calc), Mg/m^3	1.884	1.300	1.992
Abs. coefficient, mm^{-1}	2.959	0.078	4.923
F(000)	2328	852	1748
Crystal size, mm^3	0.49x0.12x0.10	0.35x0.21x0.05	0.32 x 0.07 x 0.03
Theta range, °	3.53 to 26.37	2.74 to 28.28	2.91 to 26.37
Reflections collected	10798	18591	23189
Indpt reflections (R_{int})	4344 (0.0365)	2915 (0.0469)	6117 (0.0742)
Completeness, %	99.1	99.9	99.8
Absorption correction	Multi-scan	Multi-scan	Multi-scan
Max. and min. transmission	0.713 and 0.494	1.0 and 0.744	1.0 and 0.661
Refinement method	F^2	F^2	F^2
Data /restraints/parameters	4344 / 0 / 262	2915 / 0 / 282	6117 / 0 / 435
Goodness-of-fit on F^2	1.087	1.063	1.145
R1, wR2 [I>2σ(I)]	0.0445, 0.1033	0.0458, 0.1139	0.0496, 0.0759
R1, wR2 (all data)	0.0548, 0.1096	0.0597, 0.1203	0.0585, 0.0789
Flack's parameter			-0.002(8)
Residual density, e.$Å^{-3}$	0.754 / -1.762	0.443 and -0.324	2.094 / -3.807

Table S2: Bond lengths [Å] and angles [°].

Compound **5**			
I(2)-I(2)[i]	2.8272(11)	O(1)-C(1)	1.198(5)
N(1)-C(15)	1.339(6)	N(2)-C(25)	1.340(6)
N(1)-C(11)	1.344(6)	N(2)-C(21)	1.351(6)
N(3)-C(31)	1.338(5)	N(4)-C(41)	1.335(6)
N(3)-C(35)	1.339(5)	N(4)-C(45)	1.352(5)
N(4)-C(2)	1.472(5)	C(23)-C(24)	1.379(8)
C(1)-C(35)	1.493(6)	C(24)-C(25)	1.385(7)
C(1)-C(2)	1.537(6)	C(31)-C(32)	1.396(6)
C(11)-C(12)	1.394(6)	C(32)-C(33)	1.386(7)
C(11)-C(21)	1.490(6)	C(33)-C(34)	1.375(7)
C(12)-C(13)	1.384(7)	C(34)-C(35)	1.393(6)
C(13)-C(14)	1.391(7)	C(41)-C(42)	1.382(6)
C(14)-C(15)	1.395(6)	C(42)-C(43)	1.377(7)
C(15)-C(31)	1.490(6)	C(43)-C(44)	1.379(7)
C(21)-C(22)	1.383(6)	C(44)-C(45)	1.374(7)
C(22)-C(23)	1.391(7)		

C(15)-N(1)-C(11)	118.0(4)	C(41)-N(4)-C(45)	121.9(4)
C(25)-N(2)-C(21)	117.0(4)	C(41)-N(4)-C(2)	120.5(4)
C(31)-N(3)-C(35)	118.2(4)	C(45)-N(4)-C(2)	117.5(4)
O(1)-C(1)-C(35)	124.2(4)	C(24)-C(23)-C(22)	118.5(5)
O(1)-C(1)-C(2)	122.3(4)	C(25)-C(24)-C(23)	118.3(5)
C(35)-C(1)-C(2)	113.4(4)	N(2)-C(25)-C(24)	124.3(5)
N(4)-C(2)-C(1)	111.3(4)	N(3)-C(31)-C(32)	121.8(4)
N(1)-C(11)-C(12)	122.9(4)	N(3)-C(31)-C(15)	116.2(4)
N(1)-C(11)-C(21)	115.0(4)	C(32)-C(31)-C(15)	122.0(4)
C(12)-C(11)-C(21)	122.1(4)	C(33)-C(32)-C(31)	118.9(4)
C(13)-C(12)-C(11)	118.4(4)	C(34)-C(33)-C(32)	119.9(5)
C(12)-C(13)-C(14)	119.5(5)	C(33)-C(34)-C(35)	117.3(4)
C(13)-C(14)-C(15)	118.1(4)	N(3)-C(35)-C(34)	123.9(4)
N(1)-C(15)-C(14)	123.1(4)	N(3)-C(35)-C(1)	114.3(4)
N(1)-C(15)-C(31)	116.2(4)	C(34)-C(35)-C(1)	121.8(4)
C(14)-C(15)-C(31)	120.7(4)	N(4)-C(41)-C(42)	119.4(4)
N(2)-C(21)-C(22)	122.2(4)	C(43)-C(42)-C(41)	120.2(5)

N(2)-C(21)-C(11)	117.5(4)	C(44)-C(43)-C(42)	119.0(4)
C(22)-C(21)-C(11)	120.3(4)	C(45)-C(44)-C(43)	119.7(4)
C(21)-C(22)-C(23)	119.7(5)	N(4)-C(45)-C(44)	119.8(4)

Symmetry code (i) :1 -x+1,y,-z+1/2

Compound **8**			
N(1)-C(15)	1.329(3)	N(2)-C(21)	1.331(3)
N(1)-C(11)	1.330(3)	N(2)-C(25)	1.335(3)
N(3)-C(31)	1.326(3)	N(4)-C(45)	1.333(3)
N(3)-C(35)	1.338(3)	N(4)-C(41)	1.340(3)
C(11)-C(12)	1.393(3)	C(21)-C(22)	1.389(3)
C(11)-C(25)	1.478(3)	C(21)-C(31)	1.482(3)
C(12)-C(13)	1.368(3)	C(22)-C(23)	1.374(4)
C(13)-C(14)	1.392(3)	C(23)-C(24)	1.373(4)
C(14)-C(15)	1.380(3)	C(24)-C(25)	1.390(3)
C(13)-C(131)	1.512(3)	C(14)-C(134)	1.490(3)
C(31)-C(32)	1.396(3)	C(41)-C(42)	1.388(3)
C(32)-C(33)	1.370(4)	C(42)-C(43)	1.371(4)
C(33)-C(34)	1.380(4)	C(43)-C(44)	1.371(4)
C(34)-C(35)	1.391(3)	C(44)-C(45)	1.378(4)
C(35)-C(41)	1.474(3)	C(131)-C(132)	1.515(4)
C(132)-C(133)	1.533(4)	C(134)-C(135)	1.564(4)
C(132)-C(135)	1.554(3)	C(135)-C(136)	1.505(4)
C(133)-C(134)	1.555(3)	C(135)-C(137)	1.519(4)

C(15)-N(1)-C(11)	117.3(2)	C(31)-N(3)-C(35)	118.66(19)
C(21)-N(2)-C(25)	118.3(2)	C(45)-N(4)-C(41)	117.2(2)
N(1)-C(11)-C(12)	122.6(2)	N(4)-C(41)-C(42)	122.4(2)
N(1)-C(11)-C(25)	116.9(2)	N(4)-C(41)-C(35)	116.5(2)
N(1)-C(15)-C(14)	124.3(2)	N(4)-C(45)-C(44)	123.9(2)
N(2)-C(21)-C(22)	122.6(2)	N(3)-C(31)-C(32)	122.4(2)
N(2)-C(21)-C(31)	116.7(2)	N(3)-C(31)-C(21)	116.5(2)
N(2)-C(25)-C(24)	122.4(2)	N(3)-C(35)-C(34)	122.4(2)
N(2)-C(25)-C(11)	116.5(2)	N(3)-C(35)-C(41)	116.3(2)
C(12)-C(11)-C(25)	120.5(2)	C(42)-C(41)-C(35)	121.0(2)
C(13)-C(12)-C(11)	119.5(2)	C(43)-C(42)-C(41)	118.9(2)
C(12)-C(13)-C(14)	118.4(2)	C(44)-C(43)-C(42)	119.4(2)

C(12)-C(13)-C(131)	123.5(2)	C(43)-C(44)-C(45)	118.2(2)
C(14)-C(13)-C(131)	118.0(2)	C(13)-C(131)-C(132)	110.5(2)
C(15)-C(14)-C(13)	117.8(2)	C(131)-C(132)-C(133)	109.2(2)
C(15)-C(14)-C(134)	125.4(2)	C(131)-C(132)-C(135)	111.5(2)
C(13)-C(14)-C(134)	116.7(2)	C(133)-C(132)-C(135)	87.61(19)
C(22)-C(21)-C(31)	120.7(2)	C(132)-C(133)-C(134)	86.35(19)
C(23)-C(22)-C(21)	118.7(2)	C(14)-C(134)-C(133)	106.6(2)
C(24)-C(23)-C(22)	119.3(2)	C(14)-C(134)-C(135)	110.3(2)
C(23)-C(24)-C(25)	118.7(2)	C(133)-C(134)-C(135)	86.49(18)
C(24)-C(25)-C(11)	121.1(2)	C(136)-C(135)-C(137)	108.5(2)
C(32)-C(31)-C(21)	121.1(2)	C(136)-C(135)-C(132)	119.5(3)
C(33)-C(32)-C(31)	118.8(2)	C(137)-C(135)-C(132)	112.1(2)
C(32)-C(33)-C(34)	119.4(2)	C(136)-C(135)-C(134)	118.1(2)
C(33)-C(34)-C(35)	118.4(2)	C(137)-C(135)-C(134)	111.8(2)
C(34)-C(35)-C(41)	121.3(2)	C(132)-C(135)-C(134)	85.33(18)

Complex **9**			
Pt(1)-N(1)	2.046(6)	Pt(1)-N(2)	1.936(7)
Pt(1)-N(3)	1.934(6)	Pt(1)-N(4)	2.052(6)
N(1)-C(11)	1.384(10)	N(2)-C(21)	1.341(9)
N(1)-C(15)	1.355(10)	N(2)-C(25)	1.360(10)
N(3)-C(31)	1.363(10)	N(4)-C(41)	1.405(10)
N(3)-C(35)	1.332(10)	N(4)-C(45)	1.339(10)
C(11)-C(12)	1.390(11)	C(21)-C(22)	1.391(11)
C(11)-C(25)	1.462(11)	C(21)-C(31)	1.481(11)
C(12)-C(13)	1.406(10)	C(22)-C(23)	1.373(12)
C(13)-C(14)	1.397(11)	C(23)-C(24)	1.394(10)
C(14)-C(15)	1.380(11)	C(24)-C(25)	1.384(10)
C(31)-C(32)	1.369(10)	C(41)-C(42)	1.356(11)
C(32)-C(33)	1.398(11)	C(42)-C(43)	1.374(11)
C(33)-C(34)	1.376(12)	C(43)-C(44)	1.401(11)
C(34)-C(35)	1.362(10)	C(44)-C(45)	1.382(11)
C(35)-C(41)	1.479(12)	C(131)-C(132)	1.540(12)
C(13)-C(131)	1.485(10)	C(14)-C(134)	1.497(12)
C(132)-C(135)	1.548(12)	C(133)-C(136)	1.561(13)
C(132)-C(133)	1.561(13)	C(133)-C(134)	1.564(13)

C(133)-C(137)	1.510(13)	C(134)-C(135)	1.540(12)
S(1)-O(11)	1.430(7)	S(2)-O(23)	1.407(6)
S(1)-O(13)	1.430(6)	S(2)-O(22)	1.412(6)
S(1)-O(12)	1.439(6)	S(2)-O(21)	1.478(7)
S(1)-C(1)	1.810(9)	S(2)-C(2)	1.776(10)
C(1)-F(12)	1.320(10)	C(2)-F(22)	1.288(11)
C(1)-F(13)	1.323(9)	C(2)-F(21)	1.308(11)
C(1)-F(11)	1.326(10)	C(2)-F(23)	1.380(14)

N(3)-Pt(1)-N(2)	82.2(3)	N(3)-Pt(1)-N(4)	80.3(3)
N(3)-Pt(1)-N(1)	162.9(3)	N(2)-Pt(1)-N(4)	162.4(3)
N(2)-Pt(1)-N(1)	80.8(3)	N(1)-Pt(1)-N(4)	116.8(3)
C(15)-N(1)-C(11)	117.9(7)	C(21)-N(2)-C(25)	125.2(7)
C(15)-N(1)-Pt(1)	129.4(5)	C(21)-N(2)-Pt(1)	116.4(6)
C(11)-N(1)-Pt(1)	112.7(5)	C(25)-N(2)-Pt(1)	118.4(5)
C(35)-N(3)-C(31)	124.7(7)	C(45)-N(4)-C(41)	118.0(7)
C(35)-N(3)-Pt(1)	120.1(6)	C(45)-N(4)-Pt(1)	129.3(6)
C(31)-N(3)-Pt(1)	115.2(5)	C(41)-N(4)-Pt(1)	112.6(5)
N(1)-C(11)-C(12)	121.6(7)	N(4)-C(41)-C(35)	113.8(7)
N(1)-C(11)-C(25)	115.0(7)	N(4)-C(45)-C(44)	122.0(7)
N(1)-C(15)-C(14)	123.3(8)		
N(2)-C(21)-C(22)	118.1(8)	N(3)-C(31)-C(32)	118.2(8)
N(2)-C(21)-C(31)	112.8(7)	N(3)-C(31)-C(21)	113.4(7)
N(2)-C(25)-C(24)	117.2(7)	N(3)-C(35)-C(34)	118.5(9)
N(2)-C(25)-C(11)	113.2(7)	N(3)-C(35)-C(41)	113.1(7)
C(12)-C(11)-C(25)	123.4(8)	C(24)-C(25)-C(11)	129.5(8)
C(11)-C(12)-C(13)	119.2(8)	C(32)-C(31)-C(21)	128.3(8)
C(14)-C(13)-C(12)	119.1(7)	C(31)-C(32)-C(33)	118.0(8)
C(14)-C(13)-C(131)	118.5(7)	C(34)-C(33)-C(32)	121.4(8)
C(12)-C(13)-C(131)	122.4(8)	C(35)-C(34)-C(33)	119.2(9)
C(15)-C(14)-C(13)	118.8(7)	C(34)-C(35)-C(41)	128.4(8)
C(15)-C(14)-C(134)	122.4(8)	C(42)-C(41)-N(4)	121.5(8)
C(13)-C(14)-C(134)	118.7(7)	C(42)-C(41)-C(35)	124.7(7)
C(22)-C(21)-C(31)	129.1(8)	C(41)-C(42)-C(43)	120.0(8)
C(23)-C(22)-C(21)	118.9(8)	C(42)-C(43)-C(44)	119.1(8)
C(22)-C(23)-C(24)	121.2(8)	C(45)-C(44)-C(43)	119.2(8)
C(25)-C(24)-C(23)	119.3(8)		

C(13)-C(131)-C(132)	110.7(7)	C(137)-C(133)-C(134)	121.2(8)
C(131)-C(132)-C(135)	108.9(7)	C(136)-C(133)-C(134)	109.5(8)
C(131)-C(132)-C(133)	109.9(7)	C(132)-C(133)-C(134)	87.3(7)
C(135)-C(132)-C(133)	85.7(8)	C(14)-C(134)-C(135)	104.9(7)
C(137)-C(133)-C(136)	108.0(8)	C(14)-C(134)-C(133)	107.5(8)
C(137)-C(133)-C(132)	118.5(9)	C(135)-C(134)-C(133)	85.9(7)
C(136)-C(133)-C(132)	111.1(8)	C(134)-C(135)-C(132)	88.6(7)
O(11)-S(1)-O(13)	114.9(4)	O(23)-S(2)-O(22)	119.9(5)
O(11)-S(1)-O(12)	113.9(4)	O(23)-S(2)-O(21)	112.4(5)
O(13)-S(1)-O(12)	116.2(4)	O(22)-S(2)-O(21)	111.6(5)
O(11)-S(1)-C(1)	103.2(4)	O(23)-S(2)-C(2)	105.7(5)
O(13)-S(1)-C(1)	103.3(4)	O(22)-S(2)-C(2)	105.2(5)
O(12)-S(1)-C(1)	102.8(4)	O(21)-S(2)-C(2)	99.5(6)
F(12)-C(1)-F(13)	106.9(7)	F(22)-C(2)-F(21)	110.2(10)
F(12)-C(1)-F(11)	106.1(7)	F(22)-C(2)-F(23)	105.8(10)
F(13)-C(1)-F(11)	106.4(8)	F(21)-C(2)-F(23)	103.5(9)
F(12)-C(1)-S(1)	111.5(6)	F(22)-C(2)-S(2)	114.9(7)
F(13)-C(1)-S(1)	114.0(6)	F(21)-C(2)-S(2)	113.4(8)
F(11)-C(1)-S(1)	111.4(6)	F(23)-C(2)-S(2)	108.2(8)

References for X-ray analysis

1 Altomare A., Burla M. C., Camalli M., Cascarano G. L., Giacovazzo C., Guagliardi A., Moliterni A. G. G., Polidori G., Spagna R., *J. Appl. Cryst.* **1999**, *32*, 115-119.

2 Sheldrick G. M., *Acta Cryst. A*, **2008**, *A64*, 112-122.

3 Farrugia L. J., *J. Appl. Cryst.*, **1997**, *30*, 565-566

4 Burnett M. N., Johnson C. K., *ORTEPIII*. Report ORNL-6895, Oak Ridge National Laboratory, Tennessee, USA, 1996.

5 Etter, M.C., MacDonald, J.C., Bernstein, J. *Acta Cryst.*, **1990**, *B46*, 256-262

6 Bernstein, J., Davis, R. E., Shimoni, L., Chang, N.-L. *Angew. Chem Int. Ed. Engl.*, **1995**, *34* , 1555-1573.
